# Cutaneous nerve fields of the anteromedial lower limb—Determination with selective ultrasound‐guided nerve blockade

**DOI:** 10.1002/ca.23582

**Published:** 2020-02-29

**Authors:** Georg Riegler, Christopher Pivec, Suren Jengojan, Johannes A. Mayer, Christoph Schellen, Siegfried Trattnig, Gerd Bodner

**Affiliations:** ^1^ PUC Private Ultrasound Center Graz Lassnitzhöhe Austria; ^2^ Department of Biomedical Imaging and Image‐guided Therapy Medical University of Vienna Vienna Austria; ^3^ PUC Private Ultrasound Center Wien Wien Austria; ^4^ Division of Plastic and Reconstructive Surgery, Department of Surgery Christian Doppler Laboratory for Restoration of Extremity Function, Medical University of Vienna Vienna Austria; ^5^ Department of Radiology Krankenanstalt Rudolfstiftung Vienna Austria; ^6^ Private Practice Vienna Austria

**Keywords:** anesthesia, lower extremity, neuralgia, pain, peripheral nerves, ultrasonography

## Abstract

**Background:**

This study aimed to determine the peripheral cutaneous nerve fields (CNF), their variability, and potential overlap by selectively blocking the intermediate (IFCN) and medial (MFCN) femoral cutaneous nerves and the infrapatellar branch of the saphenous nerve (IPBSN) in healthy volunteers.

**Methods:**

In this prospective study, ultrasound‐guided nerve blockades of the IFCN, MFCN, and IPBSN in 14 healthy volunteers were administered. High‐frequency probes (15–22 MHz) and 1 ml of 1% lidocaine per nerve were used. The area of sensory loss was determined using a pinprick, and all fields were drawn on volunteers' skin. A three‐dimensional (3D) scan of all lower limbs was obtained and the three CNF and their potential overlap were measured.

**Results:**

The mean size of innervation areas showed a high variability of peripheral CNF, with 258.58 ± 148.26 mm^2^ (95% CI, 169–348.18 mm^2^) for the IFCN, 193.26 ± 72.08 mm^2^ (95% CI, 124.45–262.08 mm^2^) for the MFCN, and 166.78 ± 121.30 mm^2^ (95% CI, 94.1–239.46 mm^2^) for the IPBSN. In 11 volunteers, we could evaluate an overlap between the IFCN and MFCN (range, 4.11–139.68 ± 42.70 mm^2^), and, in 10 volunteers, between the MFCN and IPBSN (range, 11.12–224.95 ± 79.61 mm^2^). In only three volunteers was an overlap area found between the IFCN and IPBSN (range, 7.46–224.95 ± 88.88 mm^2^). The 3D‐scans confirmed the high variability of the peripheral CNF.

**Conclusions:**

Our study successfully determined CNF, their variability, and the overlap of the MFCN, IFCN, and IPBSN in healthy volunteers. Therefore, we encourage physicians to use selective nerve blockades to correctly determine peripheral CNF at the anteromedial lower limb.

## 1 INTRODUCTION

**FIGURE 1:**
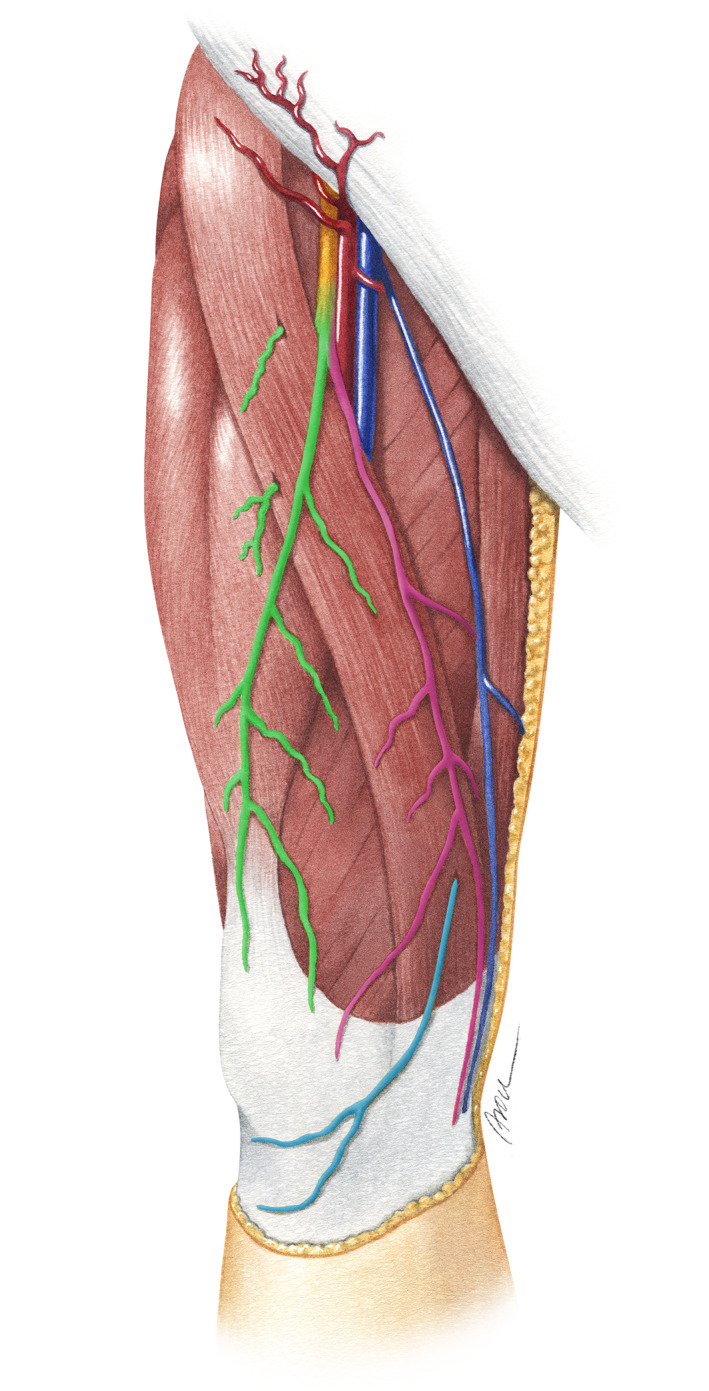
Illustration of the course of the intermediate (green) and medial (red) femoral cutaneous nerves and the infrapatellar branch of the saphenous nerve (blue) [Color figure can be viewed at wileyonlinelibrary.com]

Soft tissue trauma of the anteromedial lower limb, whether iatrogenic (e.g., knee surgeries) or traumatic (e.g., penetrating trauma), is often associated with injuries to the cutaneous nerves of this region, namely, the femoral cutaneous nerves and the infrapatellar branches of the saphenous nerve (Gage, McIlvain, Collins, Fields, & Comstock, 2012; Ginanneschi, Filippou, Frediani, & Rossi, 2013; Laffosse, Potapov, Malo, Lavigne, & Vendittoli, 2011; Pivec et al., 2018; Sundaram, Ramakrishnan, Harvey, & Parkinson, 2007; Figure 1). Due to a high nerve variability (Kerver, Leliveld, den Hartog, Verhofstad, & Kleinrensink, 2013; Mochizuki, Akita, Muneta, & Sato, 2003; Thiel, 1999), and therefore, possible different peripheral cutaneous nerve fields (CNF), the level of destruction may be appreciated incorrectly at the time of trauma. Thus, patients may sometimes be misdiagnosed and receive unnecessary treatments (Kehlet, Jensen, & Woolf, 2006).

The classical teaching about CNF covered by cutaneous nerves at the anteromedial limb in most cases is limited by several factors. A knowledge of the CNF at the anteromedial lower limb is based on anatomical textbooks (Ladak, Tubbs, & Spinner, 2014; Standring & Gray, 2008) or observational studies of hypoesthesia after several surgeries (Cohen et al., 2017; Tanavalee, Limtrakul, Veerasethsiri, Amarase, & Ngarmukos, 2016). These descriptions lack differentiation between distinct cutaneous nerve branches (e.g., medial or intermediate femoral cutaneous nerves), do not describe the possible overlap of CNF, and do not distinguish the possible variabilities. Hence, it is urgent that better ways be found to understand these differences, and thus, enhance the correct localization of CNF at the anteromedial lower limb. This may increase the correct identification of the level of nerve damage, and consequently, avoid insufficient treatment and the chance of chronic neuropathic pain. Further, accurate localization of the CNF may increase our basic knowledge about the CNF and the possible variabilities.

High‐resolution ultrasound (HRUS) has been shown to be a precise tool with which to correctly visualize the aforementioned nerve branches (Le Corroller, Lagier, Pirro, & Champsaur, 2011; Pivec et al., 2018). Moreover, in addition to visualization, HRUS is an appropriate method with which to selectively block small cutaneous nerve branches (Hasenkam, Hoy, & Soeding, 2017; Lieba‐Samal et al., 2015).

Therefore, in the present study, we hypothesized that selective nerve blockades of the intermediate (IFCN) and medial (MFCN) femoral cutaneous nerves and the infrapatellar branch of the saphenous nerve (IPBSN) can depict the CNF. We wished to determine the CNF (area of sensory loss), their variability, and potential overlap by selective injection (blockades) of local anesthetics around these branches under ultrasound guidance in healthy volunteers.

## 2 MATERIAL AND METHODS

### 2.1 Study approval

This single center, prospective study was approved by the local Institutional Review Board of the Medical University of Vienna (EC‐number 1377/2016). The study was performed in accordance with the World Medical Association Declaration of Helsinki.

### 2.2 Volunteers

Healthy volunteers were recruited via notices at the Department of Biomedical Imaging and Image‐guided Therapy of the University of Vienna and word‐of mouth acquisition. Written, informed consent was obtained from all volunteers. Inclusion criteria were age over 18, and exclusion criteria were any known polyneuropathy, myopathy, chronic disease that could cause peripheral neuropathy, current or lower limb pain, previous lower limb surgeries, numbness, hypoesthesia/paresthesia around the knee, previous use of any local anesthetics of any serotype for any reason within 1 year prior to enrollment, and potential contraindications associated with local anesthetic administration. Volunteers' history, medications, current age, size, and weight were assessed before the examination.

### 2.3 Ultrasound technique and interventions

A GE LOGIQ E9 and a GE LOGIQ e (GE Healthcare, Wauwatosa) ultrasound platform with high‐frequency probes (GE ML 6‐15‐D, GE L8‐18i‐D, GE L10‐22‐RS) were used for HRUS examinations. One radiologist with more than 6 years of experience carried out all examinations and interventional procedures (G.R.).

Ultrasound scans for the nerve branches on volunteers' right thighs were performed in accordance with the previously described techniques by *Pivec et al. (femoral cutaneous nerves; Pivec et al*., 2018) *and Riegler et al. (infrapatellar branch of the saphenous nerve; Riegler et al*., 2018) and followed a standardized assessment protocol. All participants were examined in the supine position, with the knees extended. For the IPBSN, the ultrasound examination started with a transverse view of the sartorius muscle and vastus medialis muscle, approximately 10–15 cm above the knee joint line. The sartorius muscle was then carefully screened distally until a tubular structure that pierced the fascia lata overlying the sartorius muscle, and the nerve lying in a fat pad, was assumed to be the IPBSN. To correctly assign the IPBSN to the origin from the saphenous nerve, the former was followed proximally until its origin. For the MFCN and IFCN, ultrasound examinations started with a transverse view at the middle/distal third of the anterior thigh superficial to the sartorius vastus medialis and rectus femoris muscles. The subcutaneous fat overlying these muscles was then carefully screened distally and proximally for tubular, in contrast to fat, hyperechoic structures that were presumed to be the MFCN and the IFCN. The IFCN was explored at the level overlying the vastus medialis, the rectus femoris, and, sometimes, the sartorius muscle, whereas the MFCN was explored further medially in the region of the vastus medialis and sartorius muscle. If detected, the branches were followed proximally to assess the origin from the femoral nerve so as not to confound the branches with the lateral femoral cutaneous nerve. The branches were then screened for the best locations for infiltration before splitting into further branches. To avoid a concomitant blockade of surrounding nerves, tissue layers (nerves running in fascial layers or fat pads) approximately 1–3 cm away from the origin of the nerve branches were used as blockade locations. Every nerve branch was infiltrated on a different day (in total, three consecutive days), beginning with the IFCN on Day 1, followed by the IPBSN on Day 2, and ending with the MFCN on Day 3.

**FIGURE 2:**
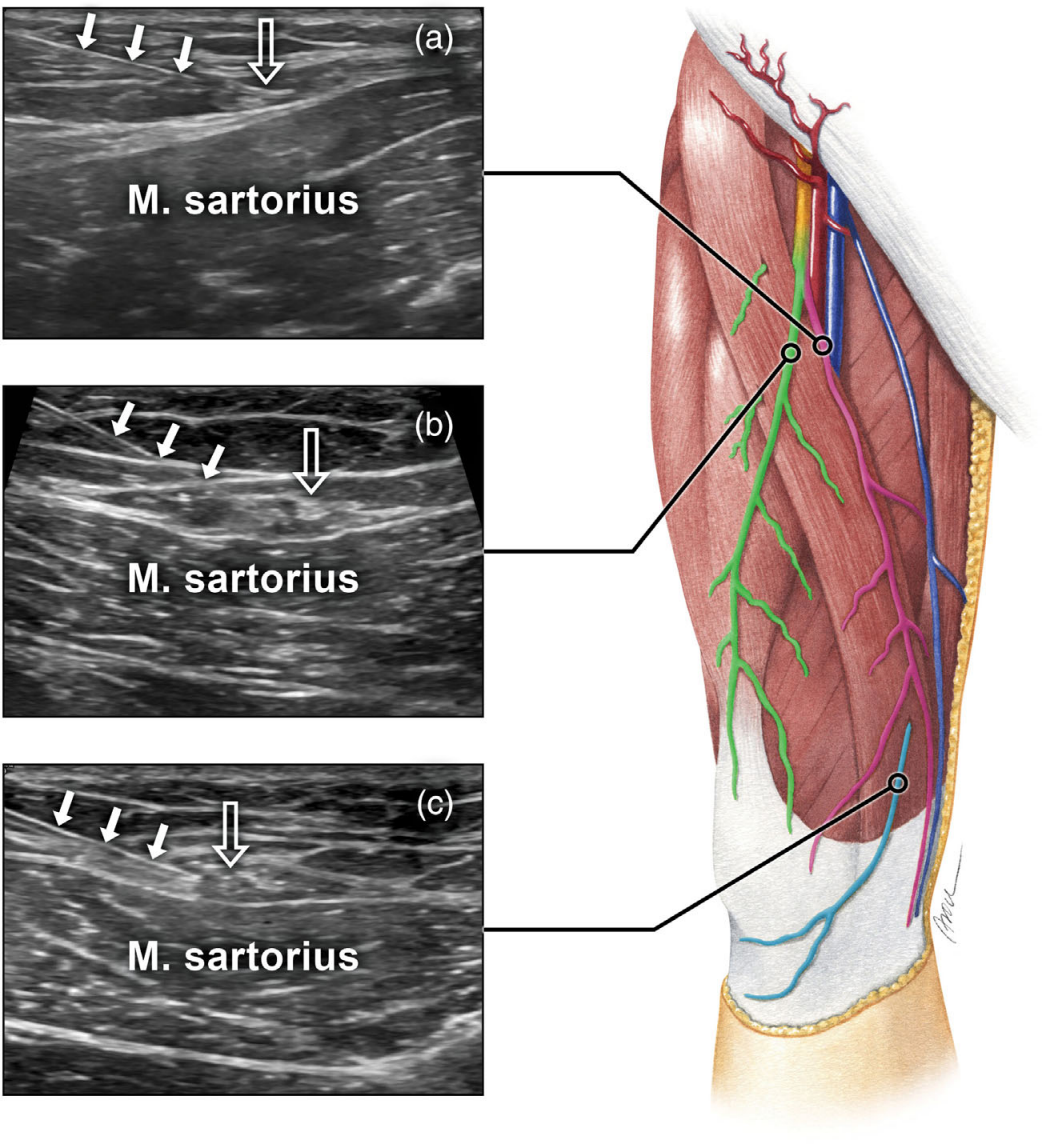
Examples of ultrasound‐guided nerve blockades of the intermediate femoral cutaneous nerve (a), the medial femoral cutaneous nerve (b), and the infrapatellar branch of the saphenous nerve (c) [Color figure can be viewed at wileyonlinelibrary.com]

The technique used to blockade the nerve branches was as follows. For all injections, the skin surrounding the needle entry point was cleaned with betadine solution. Local anesthetic administration was performed with a lateral‐to‐medial or medial‐to‐lateral approach (depending on the nerve location) using a 23‐gauge, 1.5–3.0‐in. needle (depending on the muscle size and depth). Of note, 1 cc of lidocaine (Xylanaest purum 2%, Gebro Pharma GmbH, Austria) was injected adjacent to the nerve. All interventions were accomplished using a free‐hand technique. The radiologist oriented the needle‐syringe parallel to the transducer's imaging plane and angled at approximately 45° relative to the transducer's footprint. The needle was slowly advanced under permanent ultrasound guidance. Once the needle tip was seen adjacent to the nerve, lidocaine was delivered under real‐time monitoring. The depth of infiltration (needle tip) was measured on the ultrasound image and the side of infiltration was marked on the volunteers' skin. All injection sites were screened for hematoma or other structural alterations 10 min after injection. Finally, all subjects were asked to report all adverse events during the study by completing a checklist that included: pain at the injection site lasting more than 3 days; large bruise or blood clot at the injection site; uncontrolled bleeding at the injection site; increase of pain, skin rash, or itchiness; and fever or flu‐like symptoms. Figure 2 provides examples of ultrasound‐guided nerve blockades of all three nerves.

### 2.4 Sensory blockade evaluation and drawing of innervation areas

The area of sensory loss was assessed by an independent investigator (C.S.) every 5 min for the first 30 min. The blocked area was tested using a pinprick and compared with the contralateral side. The response was scored with the following scale: 0 = *no perception*; 1 = *reduced sensation*; and 2 = *normal sensation compared with the other side*. Finally, a sensory map was drawn with a surgical marker on patients' skin relative to the marked point, which represented Grades 0 and 1 on the scale. For that reason, three different colors were used: green = IFCN; blue = IPBSN; and red = MFCN.

### 2.5 Post‐processing and 3D innervation mapping

A hand‐held three‐dimensional (3D) scanner system (3D Systems 350470 Sense2 3D‐Scanner) was used to obtain photo‐textured 3D surface models of the examined lower limbs. The scanner is not certified for medical use and has not been validated or proven for such purposes. Scans of all volunteers' lower limbs were performed along a circular path around the longitudinal axis of standing subjects with the help of a tripod‐based guidance system. All scans were performed on Day 3 after all CNF were drawn on volunteers' skin. If necessary, scans were carried out in multiple rows and the resulting 3D models were merged in postprocessing. Postprocessing of the surface models was performed with Cinema 4D R18. To confirm the accuracy of the surface models and to determine the reliability of the 3D scanner, distances between multiple control points were measured on the lower limbs and verified in the 3D models. For that reason, 20–40 cm lines were drawn on a healthy volunteer who was not taking part in the study. In vivo measurements were matched with those obtained by the 3D scan of the volunteer. Measurements deviated by less than 1 mm. Finally, all scanned CNF and their overlap were measured.

### 2.6 Statistical analysis

A statistician performed all statistical computations using IBM SPSS Statistics for Windows Version 24.0.0.2 (IBM, Armonk, NY). Metric data (size of CNF and size of overlap areas) are presented as mean ± *SD*, and range (minimum to maximum). In addition, 95% confidence intervals (CI) were calculated. A Mann–Whitney *U* test was used to compare size differences of CNF between male and female volunteers. Correlation of mean CNF values with BMI was calculated using the Pearson correlation coefficient (R). A *p* value equal to or below 5% was considered to indicate significant results.

## 3 RESULTS

### 3.1 Volunteers

Five females and nine males (mean age, 25 years; age range, 23–31 years) were included in the study. Volunteers' mean size and weight was 175.4 cm (range, 165–189 cm) and 70.4 kg (range, 56–88 kg), respectively. The mean body mass index was 22.79. One prior Osgood Schlatter and one prior posterior cruciate ligament surgery on two patients' left side were reported. None of the volunteers were medically treated at the time of the study. No side effects or adverse effects of the interventions were reported.

### 3.2 Intervention

The size of each volunteer's CNF and overlap areas are presented in Tables 1 and 2. In one volunteer (Nr. 5), we were unable to detect the MFCN, and, in another volunteer (Nr. 3), we were unable to detect the IFCN sonographically, so no CNF could be determined. The mean size of innervation areas was 258.58 ± 148.26 mm^2^ (95% CI, 169–348.18 mm^2^) for the IFCN, 193.26 ± 72.08 mm^2^ (95% CI, 124.45–262.08 mm^2^) for the MFCN, and 166.78 ± 121.30 mm^2^ (95% CI, 94.1–239.46 mm^2^) for the IPBSN. In 11 volunteers, we could evaluate an overlap between the IFCN and MFCN (range, 4.11–139.68 ± 42.70 mm^2^), and, in 10 volunteers, between the MFCN and IPBSN (range, 11.12–224.95 ± 79.61 mm^2^). There was an overlap area between the IFCN and IPBSN in only three volunteers (range, 7.46–224.95 ± 88.88 mm^2^).

**TABLE 1:** Demographic characteristics and peripheral cutaneous nerve fields of volunteers' right lower limbs in square millimeters

Volunteer no.	Age (year)	Sex	IFCN	MFCN	IPBSN
1	24	M	325.07	101.55	224.93
2	27	M	590.72	399.99	431.16
3	27	F		200.99	267.04
4	25	M	330.85	158.64	98.08
5	23	F	109.49		46.40
6	27	M	209.62	226.33	174.59
7	24	F	291.77	100.49	106.74
8	23	F	125.90	129.21	62.20
9	31	M	296.36	436.93	59.80
10	23	M	140.03	173.46	280.97
11	24	M	234.88	213.07	359.24
12	26	M	468.24	80.60	105.41
13	23	F	90.35	68.10	45.09
14	24	M	148.32	223.07	73.25

Abbreviations: IFCN, intermediate femoral cutaneous nerve; IPBSN, infrapatellar branch of the saphenous nerve; MFCN, medial femoral cutaneous nerve; No., number.

**TABLE 2:** Demographic characteristics and overlap of peripheral cutaneous nerve fields of volunteers' right lower limbs in square millimeters

Volunteer no.	Age (year)	Sex	IFCN MFCN	MFCN IPBSN	IFCN IPBSN
1	24	M	4.11	11.12	
2	27	M	38.64	274.86	
3	27	F		72.85	
4	25	M	139.68	19.42	
5	23	F			
6	27	M	80.75		
7	24	F	10.70	20.38	7.46
8	23	F	34.93		
9	31	M	65.61	39.42	
10	23	M	24.32	28.33	
11	24	M	127.89	147.88	224.95
12	26	M	80.62	16.81	107.63
13	23	F			
14	24	M	79.16	31.35	

Abbreviations: IFCN, intermediate femoral cutaneous nerve; IPBSN, infrapatellar branch of the saphenous nerve; MFCN, medial femoral cutaneous nerve; No., number.

There were no significant differences between CNF in male and female volunteers for the MFCN (*p =* .106) and IPBSN (*p =* .147), whereas, for the IFCN, we observed significant differences (*p =* .019). A correlation analysis showed no correlations or significant differences between BMI and CNF (IFCN: *R* = .389, *p =* .019; MFCN: *R* = .380, *p =* .2; IPBSN: *R* = .085, *p =* .189).

Special variations were found for the IPBSN in three cases (No. 2/3/11) that demonstrated innervation from the medial proximal thigh to the medial infrapatellar region, as well as three cases (No. 2/9/10) in which innervation was supplied to half of the medial thigh, either by the IPBSN and/or the MFCN, which is normally supplied by end‐branches of the saphenous nerve. Further, the posterior popliteal fossa was innervated by the IPBSN (No. 2) and MFCN (No. 14), which is normally supported by the posterior femoral cutaneous nerve.

### 3.3 3D innervation mapping

**FIGURE 3:**
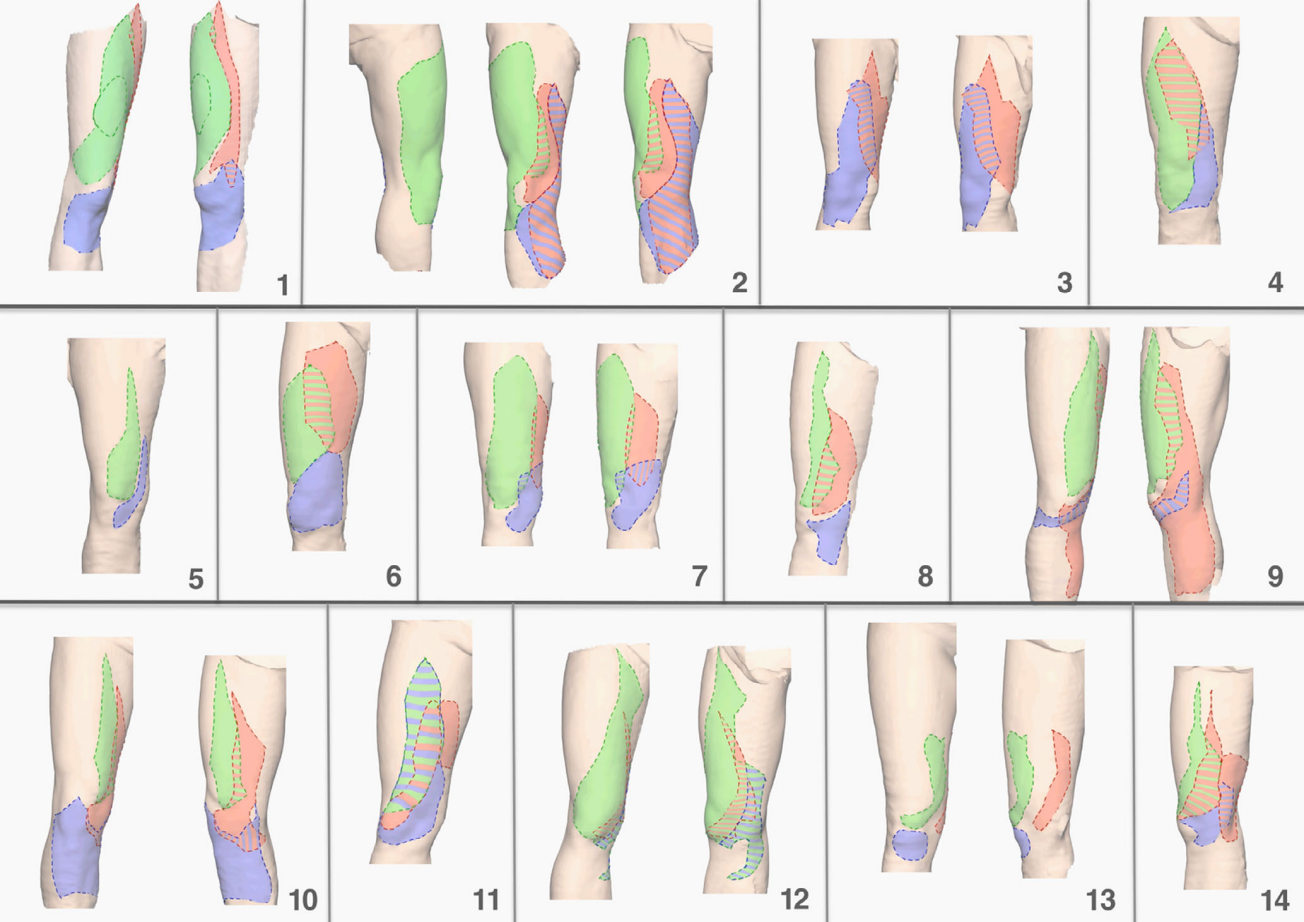
Summary of peripheral cutaneous nerve fields and their overlap (dashed lines) in all healthy volunteers (1–14) at the anteromedial lower limb. The midline of all knees in one row was matched with the others. Green: intermediate femoral cutaneous nerve; red: medial femoral cutaneous nerve; blue: infrapatellar branch of the saphenous nerve. Areas left blank suggest there was no overlap of peripheral cutaneous nerve fields [Color figure can be viewed at wileyonlinelibrary.com]

Figure 3 presents a summary of the anteromedial lower limbs of all 14‐screened volunteers. The overlap areas are presented as dashed lines.

## 4 DISCUSSION

Damage to cutaneous nerve branches at the anteromedial lower limb sometimes leads to chronic neuropathic sensations and pain due to potentially delayed diagnosis and treatment. In addition, the CNF differ among individuals, further exacerbating the difficulty of correctly locating the nerve impairment. Here, ultrasound‐guided nerve blockades were shown to be accurate in determining and differentiating between the different nerve fields of the intermediate (IFCN) and medial (MFCN) femoral cutaneous nerves and the infrapatellar branch of the saphenous nerve (IPBSN). Moreover, using ultrasound‐guided blockades, we were able to anatomically map these CNF and to show the large differences and overlap, for the first time, in human lower limbs in vivo. The anatomical basis for this work was the cutaneous nerves, which, interestingly, have been addressed differently in the past. The anatomy of the IPBSN, its course, and distribution is well known (Kalthur et al., 2015; Kartus, Ejerhed, Eriksson, & Karlsson, 1999; Kerver et al., 2013), whereas there is only a limited amount of research literature about the anterior femoral cutaneous nerves (IFCN and MFCN) exists. The description and definition for the IFCN and MFCN used in this article relies on anatomical works that differentiate the anterior femoral cutaneous nerves into two main branches (IFCN and MFCN) from which the smaller end branches arise (Anloague & Huijbregts, 2009; Gray & Clemente, 1985; Thiel, 1999). This knowledge is further supported by the actual work of Pivec et al. (2018) where this pattern was described using ultrasound to detect the nerves. Nevertheless, the course, distribution, and branching pattern seem to be highly variable and should be further addressed in future anatomical works.

Although cutaneous nerve damage at the anteromedial lower limb is known to cause neuropathic pain (Ginanneschi et al., 2013; Laffosse et al., 2011; Pivec et al., 2018; Sundaram et al., 2007), it seems to be a minor concern in our clinical routine. This may be primarily explainable by the fact that, until recently, these nerves were not accurately identifiable by imaging and only little is known about their anatomical distribution. In 2006, Lundblad, Kapral, Marhofer, and Lonnqvist (2006) first identified and blocked the main branch of the IPBSN by ultrasound. One decade later, the study group of Pivec et al. (2018) observed the different branches of the anterior femoral cutaneous nerve (IFCN and MFCN) with ultrasound. Based on these findings, our study aimed to determine the anatomical pattern of the CNF. To the best of our knowledge, until now, neither anatomical study nor anatomical textbook has described these patterns (Ladak et al., 2014; Standring & Gray, 2008). Our findings demonstrate substantial variation in innervation between individuals, which ranges between 330.90 and 386.67 mm^2^ for all the different nerves. Moreover, the observed different patterns of innervation may have a significant impact on testing these nerves in the clinical routine. With regard to the IPBSN, there were three volunteers (No. 2/3/11) who demonstrated innervation from the medial proximal thigh to the medial infrapatellar region. This is easily explainable by the origin of the branch high on the saphenous nerve. Nevertheless, it has important implications for practitioners attempting to assess IPBSN damage, as such an assessment should involve testing the whole medial lower limb. Another interesting finding was that half of the medial thigh in volunteer No. 2/9/10 was either supplied by the IPBSN and/or the MFCN, which is normally supplied by end‐branches of the saphenous nerve. To our knowledge, this is a unique finding and has further important meanings for practitioners, who should avoid misdiagnosis of pain or numbness in this area due to suspicion of saphenous nerve damage. In addition to the described variations, the most constant pattern was found for the IPBSN that always supported the infrapatellar region and the IFCN, which always supported the thigh in a midline position. Further, the posterior popliteal fossa was innervated by the IPBSN (No. 2) and MFCN (No. 14), which is normally supported by the posterior femoral cutaneous nerve (Ladak et al., 2014). Some of these findings may be explained by the variable course (Kerver et al., 2013; Pivec et al., 2018) or the communication between several nerves, of which some form plexuses around the knee (Gray & Clemente, 1985; Ladak et al., 2014; Thiel, 1999). In addition, a concomitant blockade of some adjacent nerve branches (e.g., the medial tibial branches of the saphenous nerve in case of an IPBSN blockade) may lead to such a phenomenon and represents a limitation of our study. Nevertheless, it cannot explain all our findings. For example, the MFCN in case No. 14 was blocked farther proximally where a concomitant blockade of the posterior femoral cutaneous nerve is technically impossible.

Highly variable, but constantly overlapping, areas of CNF present another important finding of our study. In about 71% (MFCN/IPBSN) and 79% (MFCN/IFCN), we could observe such an overlap for adjacent CNF. This further strengthens the theory of peripheral nerve communications and/or the presence of connections at the level of dorsal roots, dorsal root ganglia, or spinal nerves, and also, that CNF are not static (Greenberg, 2003; Ladak et al., 2014). Nevertheless, there remains much ambiguity about the CNF at the anteromedial lower limb. This article is not intended as a comprehensive work in this regard, but rather, is intended to encourage future research. A primary limitation previously, which was that cutaneous nerves were not detectable via any imaging modality until recently, has now been addressed by the excellent resolution of ultrasound (Meng et al., 2015; Tagliafico, Bignotti, Cadoni, Perez, & Martinoli, 2015). With a slight adaption of our presented assessment protocol (e.g., perhaps a slight elevation of the lidocaine dose, or an enhancement of the 3D scan), in vivo determination of CNF of a whole extremity seems to be possible in the near future. Our study has several limitations. As mentioned above, we cannot exclude concomitant blockade of different cutaneous nerves. By precisely evaluating the spread of the local anesthetic under ultrasound‐guidance and avoiding blockade of a cutaneous nerve in direct proximity to another, we tried to prevent such a mistake. In two cases, we could not find a single MFCN or IFCN. Sometimes, only one, rather than two, branches existed (Anloague & Huijbregts, 2009) which may explain Case No. 5 in which the IFCN of the CNF immediately touched the IBPSN and was not separated by the CNF of the MFCN. Nevertheless, in Case No. 3, we were unable to detect the IFCN, and therefore, no IFCN of the CNF was evaluable. Another limitation is the fact that we included only three cutaneous nerve branches and did not include the obturator or the genitofemoral nerve to evaluate the CNF of the whole anteromedial thigh. This was mainly limited by ethical considerations to avoid five nerve blockades, with all the possible side effects, on healthy volunteers. Moreover, it has to be noted that, due to the assessment protocol, to avoid concomitant blockades, and therefore, blocking the nerve rather than the origin more distally, we could not observe the most proximal part of any of the CNF in our study.

Other limitations to be noted are the small sample size and the inherent subjectivity in pinprick testing, which may lead to biases in correctly evaluating CNF.

In conclusion, our study successfully determined CNF, their variability, and overlap of the anterior femoral cutaneous nerves and the infrapatellar branch of the saphenous nerve in healthy volunteers.

## CONFLICT OF INTEREST

Dr. Georg Riegler reports a grant from the Medical Scientific Fund of the Mayor of the City of Vienna (Project number: 15200) during the conduct of the study. All other authors declare no conflict of interest.
